# Experimental Research of Bond Strength of Lightweight Aggregate Concrete to 15.7 mm Non-Pretensioned Steel Strands

**DOI:** 10.3390/ma17174361

**Published:** 2024-09-03

**Authors:** Andrzej Seruga, Rafał Stanisław Szydłowski, Łukasz Ślaga

**Affiliations:** Department of Civil Engineering, Cracow University of Technology, Warszawska 24 Street, 31155 Cracow, Poland; andrzej.seruga@pk.edu.pl (A.S.); lukasz.slaga@pk.edu.pl (Ł.Ś.)

**Keywords:** bond stress–slip relation, embedment length of steel strand, lightweight aggregate concrete, sintered fly ash artificial aggregate

## Abstract

This paper deals with the issue of the bond of concrete with the new artificial aggregate Certyd to prestressing steel strands. The solution of the problem is of great importance in the development of the use of lightweight aggregate concrete for prestressed concrete elements. Experimental research on the bond stress–slip relationship of concrete to 15.7 mm non-pretensioned steel strand was carried out. The results of bond stress–slip tests for various embedment lengths (40, 80, 120, 240, 330 and 460 mm) for test specimens made of the same lightweight aggregate concrete mixture, in which the transfer of prestressing force took place at different levels of concrete maturity (after 3, 7 and 28 days of concrete maturing), are presented. Based on the obtained results, an analytical model of the bond stress–slip relationship of lightweight aggregate Certyd concrete to 15.7 mm non-pretensioned steel strand was proposed. The tests presented demonstrated that the lightweight aggregate (Certyd) concrete is suitable for the production of pretensioned concrete elements.

## 1. Introduction

### 1.1. Lightweight Aggregate in Structural Concrete

The use of lightweight aggregate concrete (LWAC) in reinforced concrete structures, such as towering buildings, expansive bridges, and offshore platforms, is highly advantageous due to its low density, high specific strength, and dependable durability [1,2,3]. However, a significant challenge with LWAC is its distinct bond behavior compared to normal-weight concrete (NWC), primarily due to the lower strength associated with lightweight aggregates. The reduced strength of lightweight aggregates (LWA) can result in subpar bond performance for LWAC in comparison to NWC [4]. Increasing the proportion of binder materials in LWAC can enhance the strength of the mortar matrix, thereby improving bond strength [4]. Additionally, the superior interlocking effect of LWA further boosts the bond strength between reinforcing bars and concrete [5,6].

In existing investigations on the bond–slip behavior between reinforcing bars and LWAC, the expression of the bond–slip relationship is generally obtained by using segmental equations based on experimental results [7]. Key points such as peak point and residual stress of LWAC are not similar to those of NWC.

**Figure 1:**
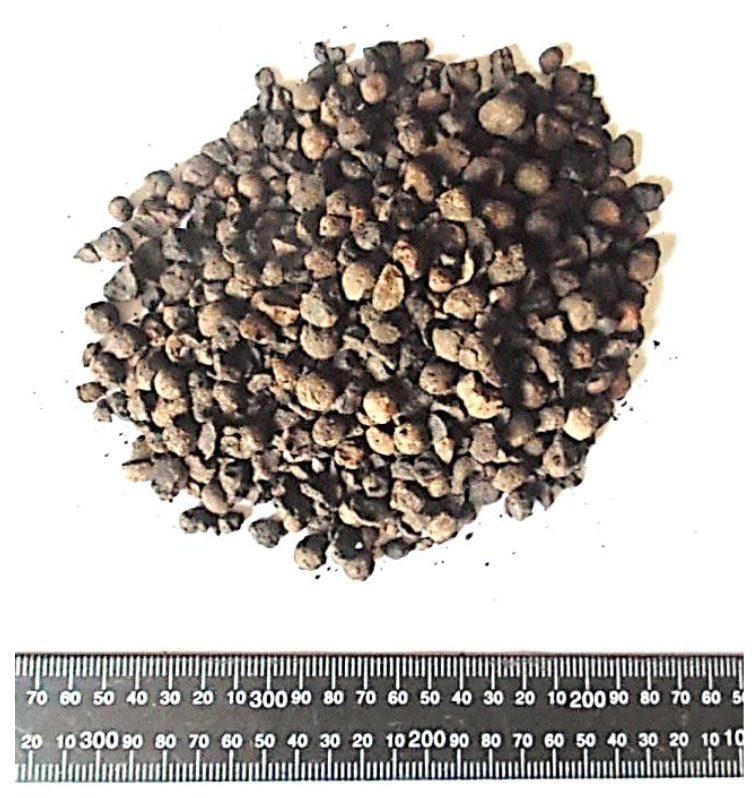
Artificial aggregate Certyd—differentiation of fraction and shape (ruler in mm) [8].

Natural lightweight aggregate includes diatomite, pumice, volcanic scoria, oil palm shells, etc. Artificial aggregates include expanded shale, expanded clay, slate, perlite, sintered fly ash and bonded fly ash. Besides these aggregates, solidified blast furnace slag is also used for the manufacture of lightweight concrete. Due to the high demand for natural aggregate at the beginning of the 21st century, the Polish Aggregates Producers Association launched the production of artificial aggregate. The use of artificial aggregate will have a positive impact on the rational management of natural resources. The production of a new Certyd artificial aggregate was launched in Poland in 2015. Certyd aggregate is produced from ash from heat and power plants deposited on heaps, which derive from the burning of hard coal. These are fly ashes from electrostatic precipitators and ash–slag mixtures from wet carrying of furnace waste. The aggregate is produced by sintering ash at a temperature of 1200 °C [8,9]. The basic parameters of the Certyd aggregate, such as bulk density, grain density, water absorption after 12h, crush resistance and frost resistance, are given in Table 1 [8,10]. A general view of the artificial aggregate Certyd is presented in Figure 1 [8].

Concretes based on Certyd lightweight aggregate are used in Poland for the production of reinforced concrete elements. For several years, the experimental research program has been carried out at Cracow University of Technology, the aim of which is to assess the possibility of using this type of aggregate for the production of prestressed concrete elements. Experimental evaluations of shrinkage, creep and prestress losses in lightweight aggregate concrete with sintered fly ash are presented in papers [8,11]. Currently, research work is being carried out aimed at exploring the possibilities of using Certyd lightweight aggregate concrete for the production of pretensioned concrete members.

The basic parameter determining the effectiveness of prestressing is the bond stress between concrete and prestressing steel tendon. There are limited experimental studies that investigated the bond stress–slip relationship for LWAC [12]. Almost all published research works concern the bond of lightweight aggregate concrete to plain and deformed bars used for the production of reinforced concrete elements.

### 1.2. Experimental Tests of Bond Stress—State of the Art

In this section, the results of selected experimental investigations on the bond strength of lightweight concretes made of the most commonly used natural and artificial aggregates reinforced with steel reinforcing bars and non-pretensioned steel strands are presented.

#### 1.2.1. Volcanic Pumice Concrete Bond to Reinforcing Bars

Pumice is a natural material of volcanic origin produced by the release of gases during the solidification of lava. Italy remains the dominant producer of pumice. Other leading countries are Chile, Ecuador, Ethiopia, France, Germany, Greece, Spain, Turkey and the United States [13,14]. The main use for pumice is as an aggregate in lightweight building blocks. Volcanic pumice (VP) has also been used as an aggregate in the production of lightweight concrete. The lack of information regarding bond characteristics of volcanic pumice concrete (VPC) is one of the main barriers to its acceptance in the construction industry. Because of limited published studies dealing with the bond characteristic of plain and deformed bars in lightweight VPC, Hossain [4] designed the experimental program to study the influence of type of concrete (NC and VPC), type of reinforcing bars (plain or deformed), embedment length and age/strength of concrete on bond strength and failure modes. The plain bars specimens had *l_emb_* = 125 mm, while the deformed bars specimens were tested at *l_emb_* of 70, 125 and 175 mm. The pull-out specimens with a cross-section of 125 × 125 mm were tested at 1, 3, 5, 7, 14, 21 and 28 days. Plain and deformed reinforcing bars had a nominal diameter of 10 mm. Both NWC and VPC mixtures were designed for a 28-day cylinder compressive strength of 30 MPa.

For deformed bars, the specimens’ load–slip relationship and modes of failures are dependent on the compressive strength/age of concrete, embedment length and type of concrete (NWC or VPC). Bond strength increases with the increase in age/compressive strength for specimens showing pull-out and splitting failure. Bond strength also decreases with the increase in embedment length for both NWC and VPC.

The average maximum bond strength determined after 28 days of concrete curing on test elements made of conventional concrete with embedment lengths of 75, 125 and 175 mm was 14.37, 10.84, 8.05 MPa, respectively. The average maximum bond strength determined after 28 days of concrete curing on test elements made of lightweight aggregate concrete with embedment lengths of 75, 125 and 175 mm was 12.50, 9.60 and 7.06 MPa, respectively. The maximum bond strength occurred at slippage in the range of 0.9–1.3 mm. *S_max_* increases as the compressive strength of concrete increases.

#### 1.2.2. Oil Palm Shell Concrete Bond to Reinforcing Bars

Mo et al. [6] carried out the investigation of the bond characteristics of lightweight oil palm shell concrete (OPSC) and normal-weight concrete (NWC) with three different strength grades of 25, 35 and 45 MPa. The uncrushed oil palm shell with a size between 2.36 and 14 mm were used in the manufacture of OPSC. In the production of NWC, crushed granite coarse aggregate of sizes between 5 and 14 mm was used. Three mix proportions for OPSC and NWC grades were designed. All the specimens were de-molded after 24 h and water-cured until the age of testing. Bond behavior was investigated using direct pull-out tests on concrete prism with a cross-section of 200 × 200 mm and 350 mm height. The dimensions were chosen to ensure splitting failure did not occur. For all tests, steel deformed bars of 12 mm diameter were used (with a yield strength of 500 MPa). The embedment length of the steel bar of 3.5ϕ (42 mm) was maintained at mid-height of the prism to ensure a uniform distribution of slip over the bond length without yielding of the reinforcing bar.

The bond test was carried out at the age of 28 days. The cube compressive strength (100 × 100 × 100 mm) of OPSC and NWC was 25.3, 35.2, 46.0 MPa and 26.8, 35.7, 42.4 MPa, respectively. The splitting tensile strength was 2.59, 2.75, 3.55 MPa and 2.71, 3.05, 3.53 MPa, respectively. The modulus of elasticity from these investigations was found in the range of 8.9, 10.7, 15.5 GPa for OPSC and 30.1, 35.0, 37.0 GPa for NWC.

The average maximum experimental bond strength of both OPSC and NWC was 15.9, 23.0, 26.2 and 8.7, 15.0, 17.7 MPa, respectively, at a slip value equal to about 1 mm. This is in agreement with the results of the research works carried out on the bond properties of LWAC, which found higher bond strength of LWAC compared to NWC [15,16]. One of the possible reasons for the higher bond strength of OPSC is the use of higher cement content in the OPSC mixes compared to the NWC mixes. This is in agreement with Bogas et al. [16], who reported that the *w/c* ratio has the greatest influence on the bond strength of concrete, regardless of the type of aggregate used.

The concrete mixes of OPSC require a higher quality of cement paste through the use of relatively higher cement content (about 200 kg higher) coupled with lower *w/c* ratio compared to NWC of similar strength grade (0.31 instead of 0.5 for concrete of 45 MPa).

#### 1.2.3. Shell Ceramsite Concrete Bond to Reinforcing Bars

In an experimental program realized by Liu et al. [3], the crushed shell ceramsite and medium sands were used as coarse and fine aggregates of LWAC, respectively, to precast test specimens. Ordinary Portland cement (P.0 42.5), first-grade fly ash and mine powder were used as supplementary cementitious material for all mixtures. Silica fume was applied for the concrete with strength grade LC60. In addition, the poly-carboxylic type superplasticizer (SP) was employed in all mixtures. Deformed bars with 12, 16 and 20 mm diameters were used in the bond test. The ratio of water to binder (*w/b*) varied from 0.26 to 0.50 in the four mixtures, corresponding to target strength classes of 30, 40, 50 and 60 MPa.

The length of the cube specimens was equal to 200 mm. To ensure the pull-out of rebar, the embedment length was maintained within five times the reinforcing diameter. The PVC tubes were used for all specimens to ensure the length of unbonded zone. The embedment length was adapted between 50 and 80 mm. Three concrete samples (100 × 100 × 100 mm) for each group were cast simultaneously with the related specimens to obtain the concrete compressive strength *f_cu_* and the splitting tensile *f_spl_* at 28-day curing age.

The real concrete compressive strength obtained for concrete LC-30, LC-40, LC-50 and LC-60 was 41.4, 51.3, 66.0 and 87.6 MPa, respectively. The splitting tensile strength resulting from the experimental test was 2.9, 3.7, 5.1 and 6.5 MPa, respectively. The bonds of LWAC from specimens labeled with LC-30, LC-40, LC-50 and LC-60 were about 16.52–17.25 MPa, 21.69–22.84 MPa, 23.11–23.82 MPa and 25.28–32.23 MPa.

It was reported by previous studies that the smaller diameter of rebar could lead to a higher bond strength [15,17,18]. In this study, the bars with a diameter of 16 and 20 mm showed similarly better bond strength compared with bars with a diameter of 12 mm. Theoretically, longer embedment length leads to a strain penetration phenomenon and this would give rise to a non-uniform distribution of strain on the reinforcing bars along the longitudinal direction, leading to a lower bond strength of concretes with longer embedment length.

#### 1.2.4. Shale Ceramsite Concrete Bond to 17.8 mm Non-Pretensioned Steel Strand

To promote the application of lightweight aggregate concrete and 1860-grade prestressing steel strand of 17.8 mm diameter in building structures, Li and Song [12] carried out research on the bonding performance of lightweight aggregate concrete and high-strength steel strand. Parameter variables, such as cover thickness embedment length, stirrup spacing and matrix material, that influence the bond anchorage performance of the 17.8 mm high-strength prestressed steel strands and lightweight aggregate concrete were examined.

Lightweight aggregate concrete (LC 40) with ordinary Portland cement (P.0 42.5), with the admixture grade II fly ash and the coarse aggregate shale ceramsite with a particle size of 5–12 mm was used. Concrete compressive strength and tensile strength evaluated on cube test samples 150 × 150 × 150 mm after 28 days of concrete maturity were 45.8 MPa (*f_c,cyl_* = 36.6 MPa) and 3.39 MPa, respectively.

Ten groups of 30 specimens were designed in this experiment. The cross-section of the specimens was 150 × 150 mm and the lengths of the specimens were 150, 250 and 300 mm, with corresponding active bond lengths of 100, 200 and 300 mm. Bond breaker with PVC casting tube of 50 mm was used (at the loaded end). The strand at the free end and the load end was extended to 100 and 350 mmm to measure the slip at both ends of the specimen.

For test specimens P_1_ (c = 26.7 mm), P_2_ (c = 44.5 mm) and P_3_ (c = 66.0 mm), the effective bond length was 100 mm. In the initial stage of the loading, slippage at the load end and free end occurred almost simultaneously, and the slippage increased uniformly. For the test specimens P_4_ (c = 66.0 mm), the effective bond length was 200 mm, and for the test specimens P_5_ (c = 66.0 mm), the effective bond length was 300 mm; all were in good shape at the initial stage of the loading, and no cracks were generated. As the load increased, the slippage began to increase. When the load was 0.15*P_u_* and 0.20*P_u_* (*P_u_* is the ultimate load), the free end of the specimen with a 200 mm and 300 mm bond length, respectively, began to slip. When the average slippage exceeded 3 mm, cracks on the surface of test specimens were observed. As the load increased, the width of the cracks continuously increased, which extended from the load end to the free end. When the average slippage reached approximately 13 mm, the load reached its limit. Thereafter, as the slippage of steel strand increased, the load began to decrease.

In order to analyze the influence of the active bond length on the value of the average maximum bond stress, the results obtained on test elements marked as P_3_ (*l_emb_* = 100 mm), P_4_ (*l_emb_* = 200 mm) and P_5_ (*l_emb_* = 300 mm) were analyzed, in which the thickness of the concrete cover was 66 mm. Li and Song [12] provided the average values of the maximum bond stress resulting from the experimental tests, which were 12.06 MPa, 11.00 MPa and 10.59 MPa. These values were calculated assuming the circumference of the steel strand *C* = πϕ. Due to the fact that the actual circumference of the steel strand is *C* = (4/3)πϕ [19], the given values after revision are 9.05 MPa, 8.25 MPa and 7.94 MPa, respectively. Assuming the compressive strength of concrete at the time of experimental tests is *f_c,cyl_* = 36.6 MPa (*w/b* = 0.27), the obtained average values of the maximum bond stress can be considered very high. It should be noted that these values were obtained with a significant slip value of 13 mm. The reason for the increased friction may be the condition of the outer surface of the steel strand (possible traces of corrosion visible in photo 7 [12]). The dirty ends of the steel strand at the free end certainly contributed to the increased friction. Photo 6 [12] shows a 100 mm section of the steel strand covered with a layer of concrete. This means that the protruding strands outside the test element were not cleaned before the experimental tests. The authors of the work did not show how to connect the sensor to measure the strand slip. It can be assumed that it was attached to the central wire in the strand.

Based on the analysis of the bond stress–slip relation graphs given in Figure 5 at [12], revised values of the average bond stress at slip s = 2.5 mm were calculated for specimens P_3_, P_4_ and P_5_. The obtained values were 7.5 MPa, 6.0 MPa and 6.0 MPa, respectively. The authors of the research claim that in the case of specimen P_3_, slippage at the loaded and free ends of the strand occurred almost simultaneously in the initial stage of loading, regardless of the thickness of the concrete cover. Therefore, it can be concluded that the adhesion was very low. In the case of specimen P_4_, the adhesive bond was *f_b,ad_* = 0.15*f_b,max_* = 1.24 MPa, while for specimen P_5_ *f_b,ad_* = 0.20*f_b,max_* = 1.60 MPa.

Morcous et al. [20] performed bond test on 58 specimens of 18 mm diameter strands in mortar and concrete. The concrete used in this test had a 1-day strength varying from 27.5 to 68.9 MPa. The average values of bond stress at slip 2.5 mm calculated for concrete specimens of *f_c_* = 44.9 MPa (*w/b* = 0.37) and *f_c_* = 68.3 MPa (*w/b* = 0.16) were equal to 4.29 MPa and 6.32 MPa, respectively. The bond stress calculated for concrete strength *f_c_* = 44.9 MPa is 28.5% lower than the bond stress determined on test elements of types P_4_ and P_5_ made of concrete on lightweight aggregate.

Based on the analysis of the results obtained from experimental tests carried out on specimens made of expanded clay aggregate concrete, reinforced with 17.8 mm non-pretensioned steel strand, it can be concluded that:

- Expanded clay aggregate concrete is characterized by a low value of adhesive bond, which increases with the increase in embedment length. For *l_emb_* = 300 mm, *f_b,adh_* = 1.60 MPa.
- The average values of the maximum bond strength of LWAC to 17.8 mm non-pretensioned steel strand were 9.05, 8.25 and 7.94 MPa for *l_emb_* = 100, 200 and 300 mm, respectively. These values were obtained with a strand slip of approximately *s* = 13 mm. The stress level in the strand for the P_5_ specimen is approximately 0.5*f_pk_*.
- The bond stress of expanded clay aggregate concrete determined at the stand slippage of *s* = 2.5 mm on a test element with *l_emb_* = 100 mm is 7.5 MPa and it is 18% higher than the bond stress determined for a test element with *l_emb_* = 400 mm made of high-strength concrete. This proves that the presented results should be treated with great caution.

### 1.3. Significant Research 

The assessment of the influence of the type of lightweight aggregate on the bond stress of concrete to a steel bar, based on other published works, is a very difficult or even impossible issue. To make this comparison, concrete mixtures must be designed and made using lightweight aggregate and normal aggregate with the same concrete compressive strength. This condition will affect the *w/b* ratio. In addition, test specimens should be made with the same embedment length and an identical cross-section, selected so that the bond is destroyed as a result of pulling out the steel bar. The type of aggregate used for LWAC and NWC significantly affects the development of the bond stress–slip relationship. Moreover, test specimens should be stored in the conditions in which the constructed structure will work. Storing test specimens in high-humidity conditions or even immersed in water may have cognitive value. However, the bond stress values obtained in this case cannot be the basis for the design principles of structural elements. Due to their smaller dimensions compared to structural elements, test specimens should be covered, for example, with foil in order to limit the impact of shrinkage resulting from faster evaporation of moisture.

In the case of the production of pretensioned concrete elements, the completed elements are covered on a prestressing bed until the prestressing force is released. The condition for releasing the prestressing force is that the concrete reaches the required compressive strength determined on cylindrical samples with a diameter of 150 mm and a height of 300 mm. Achieving concrete compressive strength of 35–40 MPa after 24 h of curing by lightweight aggregate concrete is a difficult issue. In the production plant, an accelerated concrete maturing process can be used, which is economically unjustified nowadays. An alternative solution is to modify the composition of the concrete mixture in order to lower the *w/b* ratio, for example, by using silica fume.

### 1.4. Research Gap

The fundamental condition for using lightweight concrete made of various aggregates for pretensioned elements is the knowledge of the transmission length and anchorage length (for each type of the aggregates). Existing formulas developed for normal- and high-strength concretes cannot be used to determine these parameters. The key parameter is to determine the bond stress to the prestressing stands in the transmission zone and at the anchorage length. The most accurate approach is to determine the bond stress during the production process of prestressed concrete elements, which requires a tension track—a facility not available in every research institution. However, in the laboratory, the bond stress can be determined on non-pretensioned concrete specimens. The adhesion stress obtained through this method can subsequently be used to determine the bond stress at the transmission and anchorage lengths, provided a specific correlation coefficient is applied. Studies of this type have not been conducted to date in relation to concrete on lightweight aggregate Certyd. Poland possesses substantial reserves of raw materials suitable for producing Certyd aggregate, and there is a growing interest within the construction sector to initiate the production of pretensioned concrete elements utilizing this aggregate.

The main aim of the experimental studies conducted was to determine the relationship between bond stress and slippage of Certyd lightweight aggregate concrete to a 7-wire non-pretensioned steel strand to identify the mechanisms of bond loss and to propose a corresponding calculation model. Furthermore, the experimental tests were carried out to determine if the bond stress changes over time and what the relationship between the change in the bond stress and the increase in compressive strength is. Another critical aspect addressed in this research was the determination of the reliable active bond length required to accurately assess bond stress, which is particularly significant for tests of bond strength of lightweight aggregate concrete to pretensioned steel strands.

## 2. Materials and Methods

To establish the bond stress–slip relationships for lightweight aggregate concrete (LWAC) and non-prestressed plain seven-wire steel strands, an experimental study was conducted using push-off concrete specimens. These specimens were reinforced with a steel strand measuring 15.7 mm in diameter, which was axially embedded. The experimental program considered several factors that significantly influence this relationship:

- Bond embedment length (*l_emb_* = 40, 80, 120, 240, 330 and 460 mm);
- Concrete compressive strength (for *t* = 3, 7 and 28 days).

### 2.1. Test Program

**Figure 2:**
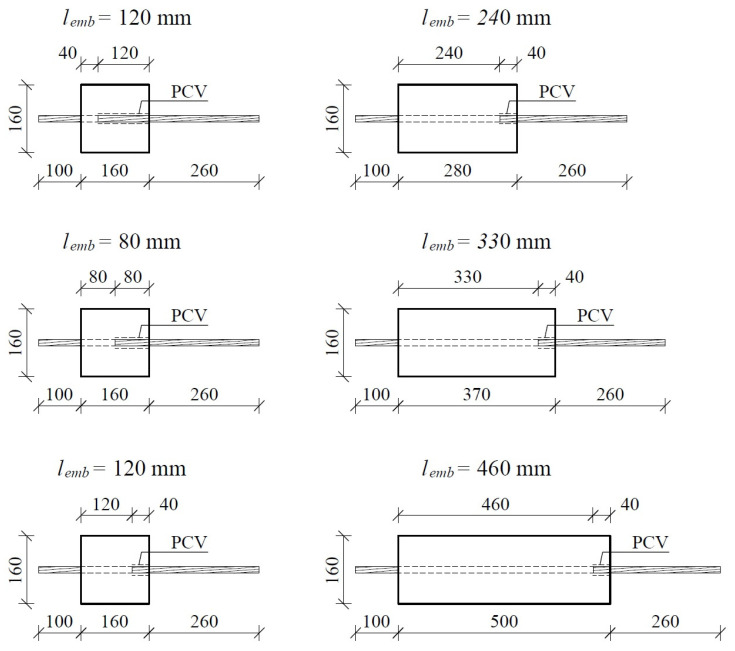
Sketch of mold with embedded strands before casting concrete.

An investigation into the bond stress between lightweight aggregate concrete and steel prestressing strands was conducted using push-off prism concrete specimens, each measuring 160 mm on all sides, with varying lengths of 160, 280, 370, and 500 mm. The specimens were evaluated after curing periods of 3, 7, and 28 days. A total of eight plywood forms for each length were fabricated, resulting in 48 forms specifically designed for this study. Prior to the casting process, the prestressing strand was secured in a horizontal orientation using rigid protective PVC tubing, which measured 120, 80, and 40 mm, corresponding to the required embedment lengths. Concrete compaction was achieved utilizing a small immersion vibrator. The arrangement of the prestressing steel strand within the individual molds for the test specimens is illustrated in Figure 2.

### 2.2. Concrete

The specimens were constructed using a specially formulated cement concrete composition, classified as C45/55. The specific ingredients required for 1 m^3^ of the concrete mix are detailed in Table 1. Rapid-hardening Portland cement 42.5 R was utilized in the formulation. All speciments were prepared in Building Materials and Structures Laboratory of Cracow University of Technology.

The specimens were produced in multiple series, each varying in their active bond length. Within each series, standard samples measuring ϕ150 × 300 mm were collected to assess the compressive strength, tensile strength, and modulus of elasticity of the concrete under compression. Following casting, all specimens and standard samples were covered with multiple layers of polyethylene sheeting to maintain stable curing conditions for a duration of seventy-two hours. After this curing period, the specimens were removed from their plywood forms, and a selection was prepared for experimental testing.

The experimental research was conducted on days 3, 7, and 28 post-curing. After demolding, all specimens and standard samples intended for testing were stored under three layers of polyethylene sheeting. At each stage of testing, the mechanical properties of the concrete were evaluated. The identification numbers of the tested specimens are provided in Table 2.

### 2.3. Steel Prestressing Strands

Steel prestressing plain seven-wire strands were used to bond test. Steel strength tests were performed with a Zwick-Roell Z1200 machine (manufacturer: Zwick-Roell, city and country of acquisition: Ulm, Germany). Tensile strength is measured automatically by an in-built force gauge. Displacement and strain are registered by an incremental extensometer synchronized with the machine software (Zwick-Roell Testexpert II, V3.2, S/N: 817411).

The extensometer’s measuring base was 180 mm. Load steering was performed at the speed of 20 MPa/s up to yield point and then it was automatically switched into displacement control at the speed of 0.003 1/s in range flow. The properties of prestressing strand used in experimental investigation are presented in Table 3.

### 2.4. Test Procedure

The initiation of production of pretensioned concrete elements necessitates the assessment of the suitability of a specific tendon type. This assessment is quantified by achieving a designated force value during tendon slippage, which occurs within a range of slip from 0.254 mm to 2.540 mm using the pull-out method [21]. The force value is influenced by various factors, including the diameter of the strand. The test specimen is composed of a precisely defined cement mortar. Alternative methodologies exist that utilize concrete mixtures instead of mortars, which concurrently elevate the required pulling force values. However, there is currently no standardized research methodology for pretensioned concrete elements that enables the determination of the bond stress between concrete and prestressing tendons, thereby hindering the evaluation of concrete’s suitability for production. The determination of bond stress is well defined for smooth and ribbed bars utilized in reinforced concrete elements, as outlined in standards such as Model Code 2010 [22]. Consequently, this study employed a modified pull-out method, wherein the concrete element is pushed-off (displaced) along non-pretensioned (or pretensioned) 7-wire steel strand.

**Figure 3:**
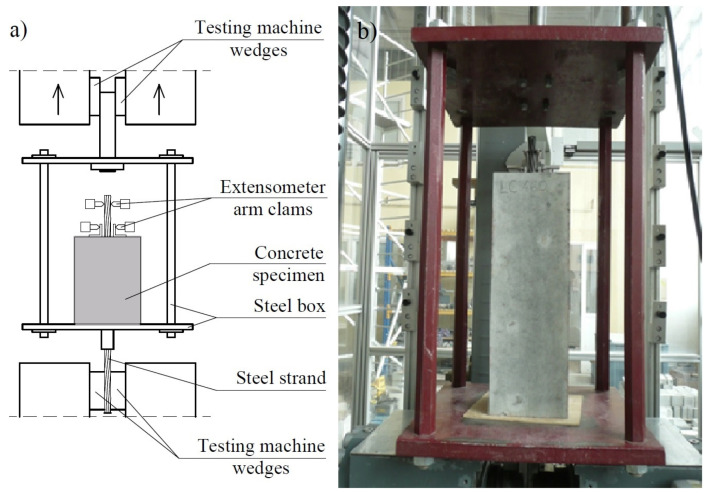
Scheme (**a**) and general view (**b**) of the concrete specimen during testing.

The specimens for testing the bond stress–slip relationship with an axially embedded steel prestressing strand were placed in a specially designed steel frame (Figure 3). The structure consists of two rigid steel plates, upper and lower, joined to each other by four steel hangers made of steel bars with a hexagonal cross-section.

**Figure 4:**
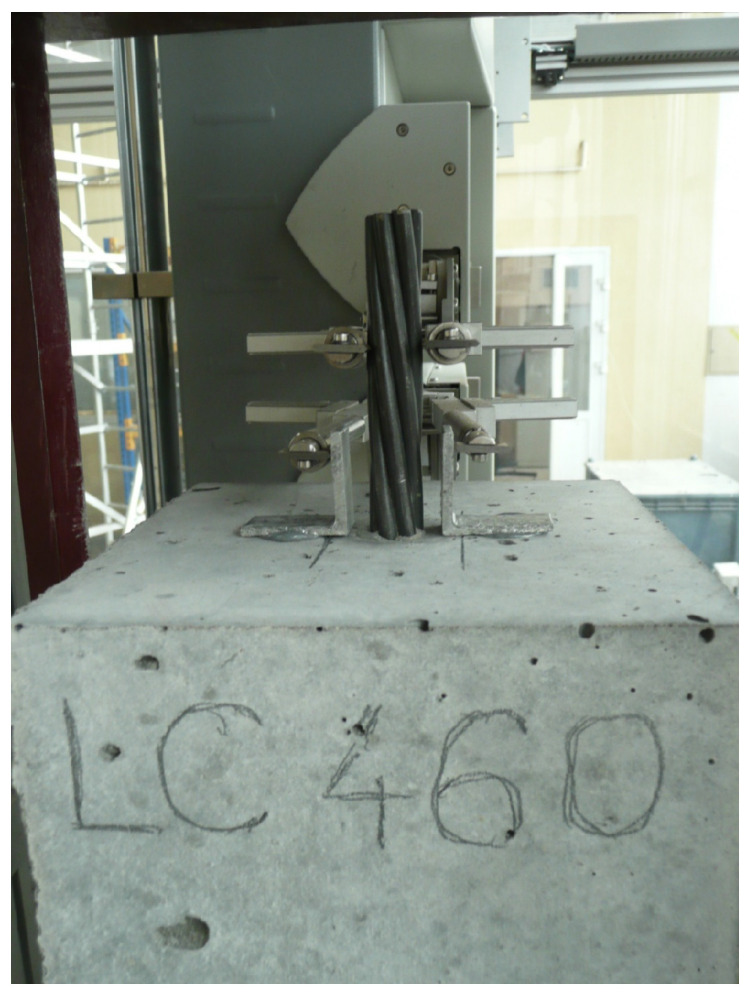
Extensometer mounted at the free end of a plain steel strand of 15.7 mm during testing.

The bottom steel plate has a centrally drilled hole through which it is possible to pass the strand coming from the concrete specimen. A longer, lower strand section is chucked in the gripping jaws of a new-generation testing machine (Zwick-Roell Z1200). The jaw pressure on the strand is 400 bar. The force from the steering mechanism lifts the frame structure, which transfers the load onto the concrete specimen through pressure on the bottom surface. The pushing load is controlled by displacement with a loading rate of 0.01 mm/s. The force value is continuously digitally registered with the help of the registering system. The initial force value is 1 kN. Relative displacement for the given force is measured by a measured system of two arms of the incremental extensometer. The upper arms register the displacement of the free end steel strand, and the lower arms register the displacement of the aluminum angles glued to the concrete surface with regard to their original location (Figure 4). The final value displayed on the computer screen is the relative displacement of two materials. The margin of error of the extensometer reading is 0.12 μm. This research was carried out until the displacement value set in the program, i.e., 10 mm, was reached.

## 3. Test Results

A total of 93 specimens of lightweight aggregate concrete, categorized as 30, 34, and 29 after 3, 7, and 28 days, respectively, were subjected to testing with non-pretensioned steel strands in the Laboratory of the Institute of Materials and Building Structures (refer to Table 2).

The compressive strength of the concrete was measured after 3, 7, and 28 days and it amounted to 35.6 MPa, 45.2 MPa, and 55.3 MPa, respectively. The modulus of elasticity was determined to be 21,560 MPa, 22,315 MPa, and 24,200 MPa for the same time intervals.

**Figure 5:**
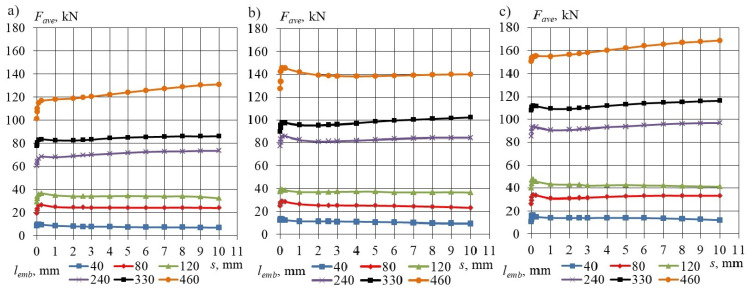
Extensometer mounted at the free end of a plain steel strand of 15.7 mm during testing. Average pushing force–slip relationship for LWAC specimen with cross-section 160 × 160 mm and non-pretensioned plain seven-wire steel strand of 15.7 mm diameter. Concrete compressive strength: (**a**) *f_cm_* = 35.6 MPa, (**b**) *f_cm_* = 45.2 MPa, (**c**) *f_cm_* = 55.3 MPa.

In analyzing the force value–slip relationship recorded electronically for each specimen, the force value indicating the loss of concrete bond to the prestressing strand was identified, along with the corresponding pushing force values at slip values of 0.01, 0.0254, 0.10, 0.254, 1.0, 2.0, 2.54, 3, 4, 5, 6, 7, 8, 9, and 10 mm. The average pushing force values associated with the specified slip values for each embedment length of a steel strand with a diameter of 15.7 mm, corresponding to concrete compressive strengths of 35.6, 45.2, and 55.3 MPa, are detailed in Table 4, Table 5 and Table 6, respectively. The distribution of average pushing force as a function of slip, defined for the specified concrete compressive strengths, is illustrated in Figure 5a–c.

The bond stress (*f_b_*) of LWAC to non-pretensioned steel strand was calculated according to the following equation:

(1) $$f_{b}=\frac{F}{C\cdot l_{emb}}$$

where the circumference *C* of the strand was obtained from relation [19]:

(2) $$C=\frac{4}{3}\pi \cdot \phi$$

**Figure 6:**
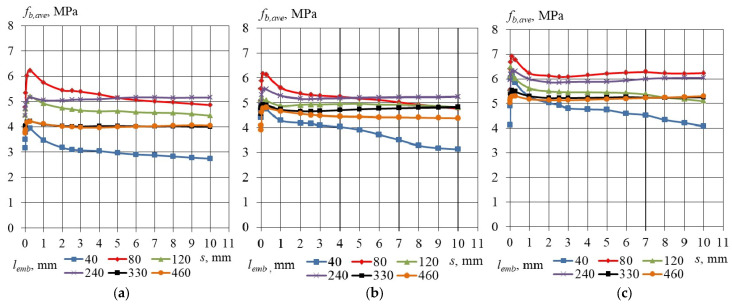
Average bond stress–slip relationship for LWAC specimen with cross-section 160 × 160 mm and non-pretensioned plain seven-wire steel strand of 15.7 mm diameter. Concrete compressive strength (**a**) *f_cm_* = 35.6 MPa, (**b**) *f_cm_* = 45.2 MPa, (**c**) *f_cm_* = 55.3 MPa.

The average bond stress values for lightweight aggregate concrete (LWAC) specimens with non-pretensioned steel strands, corresponding to concrete compressive strengths of 35.6, 45.2, and 55.3 MPa, are presented in Table 7, Table 8 and Table 9. The distribution of average bond stress as a function of slip, defined for specific concrete compressive strengths and considering the analyzed embedment lengths, is illustrated in Figure 6a–c. It is evident that the bond stress profiles for embedment lengths of 80, 120, 240, 330, and 460 mm are quite similar. However, a notable difference is observed for the embedment length of 40 mm, indicating that this length is insufficient to establish the interlocking mechanism.

## 4. Discussion of Tests Results

The experimental tests were performed to determine the feasibility of producing pretensioned concrete elements utilizing the lightweight concrete aggregate known as Certyd. The criteria for the release of the prestressing force during the manufacturing process include:

- Achieving concrete compressive strength of at least 35 MPa;
- Achieving a stress level in the strand between 0.70 and 0.75 *f_pk_*;
- Limiting strand slippage by 2–3 mm (for normal concrete).

**Figure 7:**
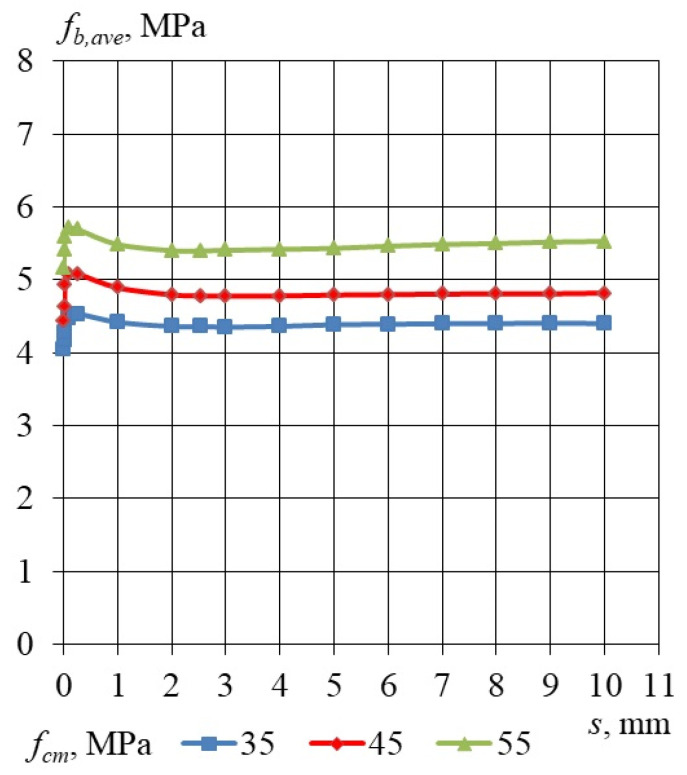
Average values of bond stress–slip relationship for LWAC to plain seven-wire steel strand of 15.7 mm diameter for *l_emb_* equal 240, 330 and 460 mm. Specimen cross-section of 160 × 160 mm.

To evaluate the results obtained, the bond stress corresponding to a slip value of 10 mm was calculated for each specimen with embedment lengths of 240, 330, and 460 mm. The average values of these calculations for concrete compressive strengths of 35.6, 45.2, and 55.3 MPa are summarized in Table 10. This table also includes the maximum bond stress values, as well as average bond stress values for slip ranges of 1–10 mm and 2–10 mm, along with the coefficients of variation (CoV). Figure 7 illustrates the average bond stress–slip relationships for the LWAC specimens, which have a cross-section of 160 × 160 mm and utilize a non-pretensioned plain seven-wire steel strand with a diameter of 15.7 mm.

Table 11 presents the average values of the maximum force required to displace the LWAC specimens from the non-pretensioned steel strand. The average maximum bond stress values for the LWAC specimens in relation to the non-pretensioned steel strand are detailed in Table 12, while Table 13 provides the average maximum stress values in the non-pretensioned steel strand. It is evident that the average maximum strand stresses were recorded in concrete specimens with compressive strengths of 35.6, 45.2, and 55.3 MPa for an embedment length of 460 mm. The ratios of strand stress to ultimate strength are 0.45, 0.51, and 0.58 for concrete compressive strengths of 35.6, 45.2, and 55.3 MPa, respectively, as shown in Table 14. Notably, all concrete specimens underwent testing with continuous monitoring of slip data, and no cracks were detected on the external surfaces of the test specimens.

**Figure 8:**
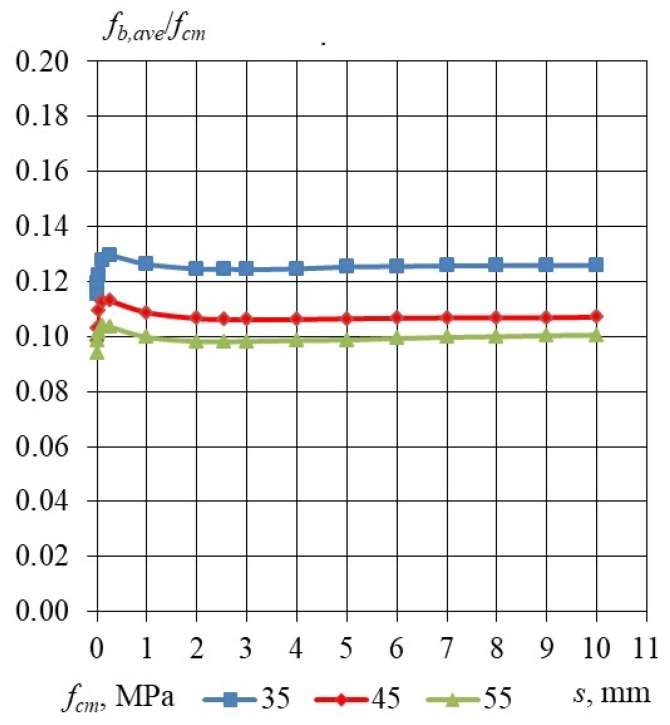
Influence of the LWAC compressive strength on the relative bond stress–slip relationship, for *l_emb_* equal to 240, 330 and 460 mm for 15.7 mm non-pretensioned steel strand. Specimen cross-section of 160 × 160 mm.

**Figure 9:**
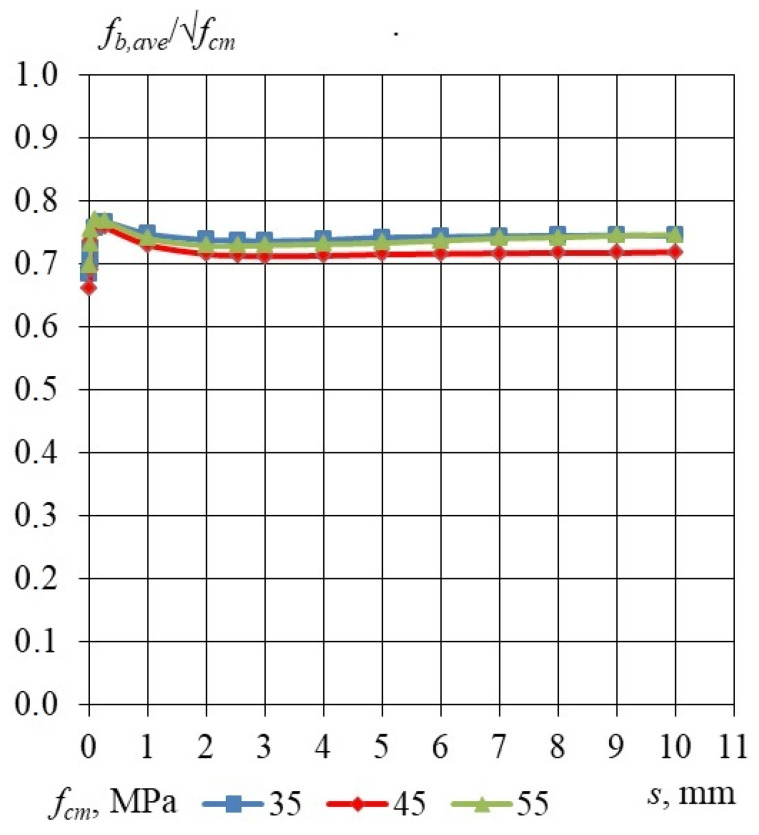
Influence of square root of the LWAC compressive strength on the relative bond stress–slip relationship, for *l_emb_* equal to 240, 330 and 460 mm for 15.7 mm non-pretensioned steel strand. Specimen cross-section of 160 × 160 mm.

Taking into consideration the results of the bond stress–slip relationship obtained for specimens with an embedment length of 240, 330 and 460 mm, the dependency of the relative bond stress *f_b_/f_cm_* and *f_b_/√f_cm_* was analyzed. The influence of the LWAC compressive strength and the square root of LWAC compressive strength on the relative bond stress–slip relationship is presented in Table 15, Table 16 and Table 17. Figure 8 shows the influence of the LWAC compressive strength on the relative bond stress–slip relationship, for *l_emb_* equal to 240, 330 and 460 mm for a 15.7 mm non-pretensioned steel strand. The influence of the square root of the LWAC compressive strength on the relative bond stress–slip relationship, for *l_emb_* equal to 240, 330 and 460 mm for a 15.7 mm non-pretensioned steel strand, is presented in Figure 9. It can be concluded that the bond strength increases proportionally to the square root of the compressive strength independently of the concrete compressive strength ranging 35–55 MPa.

Considering the bond stress–slip relationships derived from specimens with embedment lengths of 240, 330, and 460 mm, the relationship between the relative bond stress *f_b_/f_cm_* and *f_b_/√f_cm_* was examined. The effects of LWAC compressive strength and the square root of LWAC compressive strength on the relative bond stress–slip relationship are presented in Table 15, Table 16 and Table 17. Figure 8 illustrates the effect of the compressive strength of lightweight aggregate concrete (LWAC) on the relative bond stress–slip relationship for embedment lengths of 240, 330, and 460 mm, utilizing a 15.7 mm non-pretensioned steel strand. Additionally, Figure 9 depicts the influence of the square root of LWAC compressive strength on the relative bond stress–slip relationship for the same embedment lengths and steel strand specifications. It can be inferred that the bond strength exhibits a proportional increase relative to the square root of the compressive strength, regardless of the concrete compressive strength, which ranges from 35 to 55 MPa. The obtained bond stress–slip relationship profile differs from the profile presented by Li and Song [12]. The bond stress–slip relationship presented by Li and Song increased from zero stress level to a maximum value and then decreased to a residual value. The bond stress–slip relationship proposed in this study is characterized by adhesive stress (at zero slip) and residual stress (average value of bond stress for slip from 1 to 10 mm). The experimentally verified mechanism of concrete bond strength to the plain seven-wire steel strand failure is different from that which is assumed for a steel plain bar [23] as well as from that for non-pretensioned steel strand with a 15.7 mm diameter in high-performance concrete [24].

**Figure 10:**
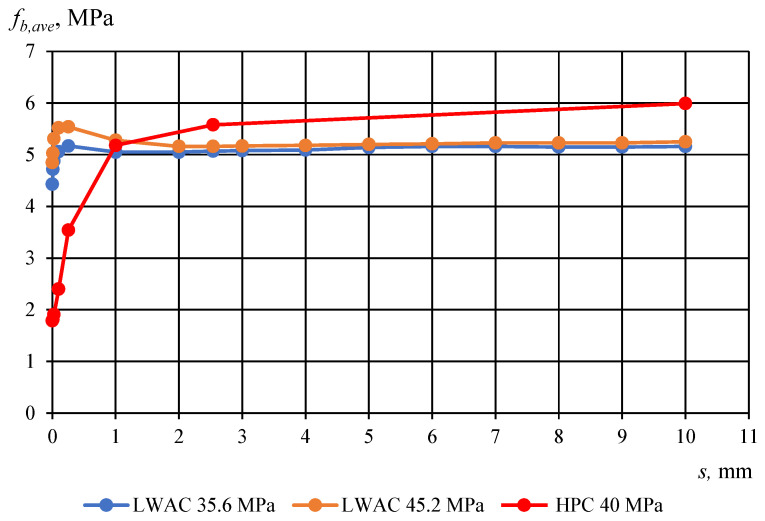
Average values of bond stress–slip relationship for LWAC and HPC (compressive strength *f_ci_* = 35–45 MPA) to plain seven-wire steel strand of 15.7 mm diameter for *l_emb_* equal 240 mm. Specimen cross-section of 160 × 160 mm.

**Figure 11:**
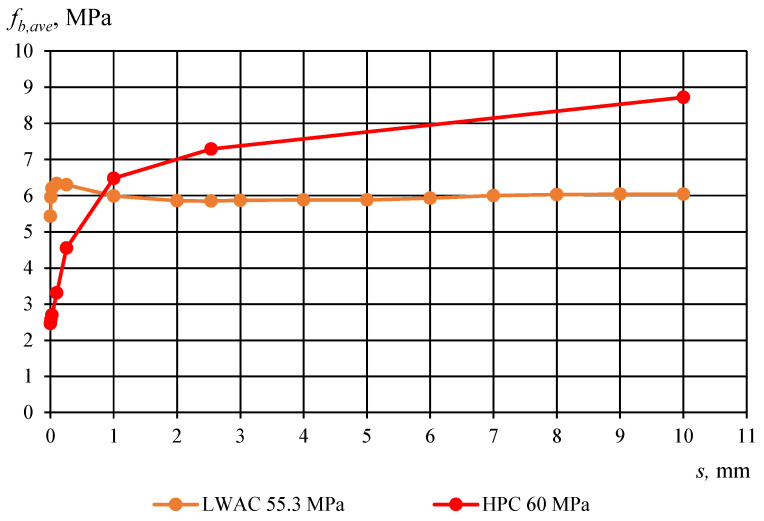
Average values of bond stress–slip relationship for LWAC and HPC (compressive strength *f_ci_* = 55–60 MPA) to plain seven-wire steel strand of 15.7 mm diameter for *l_emb_* equal 240 mm. Specimen cross-section of 160 × 160 mm.

Figure 10 and Figure 11 show the comparison between average bond stress–slip relationships for corresponding compressive strengths of LWAC and high-performance concrete (HPC) specimens, which have a cross-section of 160 × 160 mm and utilize a non-pretensioned plain seven-wire steel strand with a diameter of 15.7 mm. The two graphs illustrate that the adhesion stress for lightweight aggregate concrete (LWAC) is significantly greater compared to high-performance concrete (HPC), with LWAC exhibiting no subsequent increase in bond stress. This observation indicates an absence of the aggregate interlocking effect. Conversely, in the case of HPC, this interlocking effect is present, resulting in a consistent rise in bond stress.

Based on the results obtained from experimental tests carried out on test elements with a cross-sectional of 160 × 160 mm, the embedment length of 240, 330, 460 mm and concrete compressive strength of 35.6, 45.2 and 55.3 MPa, in this study, a computational model capturing the bond stress–slip relationship for Certyd lightweight aggregate concrete and a 15.7 mm diameter non-pretensioned steel prestressing strand was proposed.

Model for LWAC concrete bond to 15.7 mm diameter non-pretensioned steel prestressing strand:

(3) $$f_{b}=f_{b,a}+{\left(\frac{s}{s_{1}}\right)}^{\alpha }\cdot \left(f_{b,ave}-f_{b,a}\right)\;\mathrm{if}\;(0\leq s\leq s_{1})$$

$$f_{b}=f_{b,ave}\;\;\mathrm{if}\;\;(s_{1}\leq s\leq s_{10})$$

where $f_{b,a}=0.681\cdot \sqrt{f_{cm}}$; $f_{b,ave}=0.731\cdot \sqrt{f_{cm}}$.

Symbols’ explanation:

*f_b,a_*—adhesive bond;
*f_b,ave_*—average bond stress for slip from *s* = 1 mm to *s* = 10 mm;
*f_cm_*—average concrete compressive strength, range (35–55 MPa);
*s*—slip; *α*—coefficient *α* = 0.05; *s*_1_*—*1 mm slip; *s*_10_*—*10 mm slip.

## 5. Conclusions

The conclusions derived from both experimental investigations and numerical calculations regarding the bond behavior of a plain seven-wire steel strand with a diameter of 15.7 mm in lightweight aggregate concrete are as follows:

- The experimental findings indicate that bond strength exhibits a proportional increase relative to the square root of the compressive strength, regardless of the concrete compressive strength, which ranges from 35 to 55 MPa. The average bond strength for three different embedment lengths (240, 330, and 460 mm) of lightweight aggregate concrete for a 15.7 mm strand shows an upward trend with increasing concrete compressive strength over time. However, the bond strength increase, averaging 10%, is less significant than the increase in concrete compressive strength, which averages 25% over the same period.
- The adhesion bond between lightweight aggregate concrete and the non-pretensioned plain seven-wire steel strand is variable. It is influenced by both the embedment length and the concrete compressive strength, as expressed by the formula (*f_b.a_* = 0.681√*f_cm_*). The adhesion bond values are 4.05, 4.43, and 5.17 MPa for concrete compressive strengths of 35, 45, and 55 MPa, respectively. These average bond stress values were derived from experimental results for concrete specimens with embedment lengths of 240, 330, and 460 mm.
- Concerning the obtained adhesion stress values for LWAC, it is important to note that, for normal-weight concretes, the ACI standard [25] for determination of transmission length and anchorage length limits the bond stress value to 3.0 MPa.
- For the modified pull-out test method aimed at assessing the actual bond strength, lightweight aggregate concrete specimens should incorporate axially positioned non-pretensioned seven-wire steel strands with a minimum embedment length of 240 mm. A concrete specimen cross-section of 160 × 160 mm is recommended.
- The average bond strength for slip values ranging from 1 mm to 10 mm between lightweight aggregate concrete and the non-pretensioned seven-wire steel strand of 15.7 mm can be estimated using the formula *f_b,ave_* = 0.731∙√*f_cm_*. The average bond strength of lightweight aggregate concrete is only slightly influenced by the mechanical interlock mechanism. For embedment lengths of 240, 330, and 460 mm, the average bond strength remains consistent in the range of the steel strand slip from 1 to 10 mm for the analyzed LWAC compressive strengths. This average bond strength value is only 7.5% higher than the average adhesion bond strength.
- On the basis of the obtained results, the model of bond stress–slip relationship for LWAC concrete to a 15.7 mm diameter non-pretensioned steel prestressing strand was proposed (3).
- The experimentally verified mechanism of concrete bond strength to the plain seven-wire steel strand failure is different from that which is assumed for a steel plain bar [23] as well as from that for non-pretensioned steel strand with a 15.7 mm diameter in high-performance concrete [24]. This provides an opportunity for specifying the designing method of calculation of the prestressed concrete element.
- The initial phase of this research (conducted on non-pretensioned concrete specimens) demonstrates that the lightweight aggregate (Certyd) concrete is suitable for the production of pretensioned concrete elements (the stress level in the non-pretensioned strand remains below 0.6 *f_pk_*). In the next stage of the research (conducted on pretensioned concrete specimens), the influence of the Hoyer effect on the variation in bond stress, transmission length and anchorage length should be experimentally determined. This investigation will also facilitate the refinement of the *f_b_*-*s* calculation model.

## Figures and Tables

**Table 1:** Mix design for lightweight aggregate concrete.

Components		Quantities [kg/m^3^]
Rapid-hardening Portland Cement CEM I 52.5R		419
River sand (0–2) mm		703
Certyd aggregate (4–12) mm		802
Water		209
Additives	BV 18, high-performance superplasticizer, BASF	3.8
	SKY 686, h-r water reducing superplasticizer, BASF	3.8
*w/c* ratio		0.50

**Table 2:** LWAC specimens with a cross-section of 160 × 160 mm reinforced with non-pretensioned steel strand of 15.7 mm diameter.

*l_emb_* mm	Number of the Specimen Tested		
	*f_cm_*, MPa		
	35.6	45.2	55.3
40	5	5	5
80	5	6	5
120	5	5	4
240	5	6	5
330	5	6	5
460	5	6	5
*n*	30	34	29
total	93		

**Table 3:** Nominal properties of prestressing steel strand.

Geometry			Material Properties			
Strand Diameter mm	Steel Area mm^2^	Lay Length mm	Ultimate Strength *f_p_*, MPa	Breaking Strength *F_pmax_*, kN	Yield Strength *F_p01_*, kN	Modulus of Elasticity *E_p_*, MPa
15.7	150	249	1906.8	286.03	251.80	190,110

**Table 4:** Average values of force pushing the LWAC specimen off the non-pretensioned steel strand (*f_cm_* = 35.6 MPa).

*s* mm	*l_emb_*, mm											
	40		80		120		240		330		460	
	*F_ave_*, kN	*CoV* %	*F_ave_*, kN	*CoV* %	*F_ave_*, kN	*CoV* %	*F_ave_*, kN	*CoV* %	*F_ave_*, kN	CoV %	*F_ave_*, kN	*CoV* %
adhesion	8.31	20.58	21.02	20.50	32.28	13.85	68.98	23.04	85.94	19.08	113.31	21.60
0.01	9.23	14.73	25.08	21.81	35.25	12.09	73.53	19.43	86.99	18.21	114.93	19.31
0.0254	9.95	10.35	28.04	24.79	37.11	14.44	75.99	16.90	87.94	17.16	117.51	14.82
0.1	10.28	10.60	31.62	22.99	39.42	17.94	78.86	13.77	90.97	13.51	126.31	2.30
0.254	10.35	10.14	32.76	23.05	41.01	19.87	80.47	11.23	91.58	12.96	127.43	2.31
1	9.13	7.67	30.24	24.90	38.80	17.68	78.65	10.64	88.88	10.52	124.34	3.18
2	8.35	14.61	28.64	25.42	37.41	18.10	78.59	11.34	87.43	9.58	121.30	4.58
2.54	8.15	16.44	28.51	24.97	37.02	18.94	78.94	11.64	87.23	9.10	120.71	5.29
3	8.06	18.61	28.32	25.42	36.63	20.31	79.16	11.91	87.03	8.47	120.05	5.65
4	7.99	23.03	27.77	27.12	36.37	21.36	79.37	12.75	87.34	7.73	119.98	6.32
5	7.79	27.09	27.04	28.77	36.45	23.10	80.05	13.90	87.42	7.58	120.60	7.05
6	7.64	30.50	26.59	30.05	36.08	23.14	80.44	14.51	87.23	8.01	121.08	7.71
7	7.56	33.33	26.32	30.78	35.93	23.91	80.43	15.07	86.94	7.67	121.71	8.04
8	7.43	34.72	26.09	31.47	35.86	25.60	80.16	15.04	87.15	7.47	122.30	7.77
9	7.31	37.07	25.83	32.29	35.52	27.11	80.32	15.16	86.90	7.41	122.80	7.65
10	7.20	38.89	25.56	33.49	35.01	30.11	80.39	15.84	86.80	7.76	122.58	7.61
*F_max,ave_*	10.65	11.74	32.84	23.11	41.24	19.67	84.32	9.81	95.86	10.01	128.90	3.35

**Table 5:** Average values of force pushing the LWAC specimen off the non-pretensioned steel strand (*f_cm_* = 45.2 MPa).

*s* mm	*l_emb_*, mm											
	40		80		120		240		330		460	
	*F_ave_*, kN	CoV %	*F_ave_*, kN	CoV %	*F_ave_*, kN	CoV %	*F_ave_*, kN	CoV %	*F_ave_*, kN	CoV %	*F_ave_*, kN	CoV %
adhesion	11.61	10.38	26.70	25.21	38.59	14.80	76.53	21.57	98.57	14.25	118.19	21.61
0.01	12.41	11.12	29.30	20.38	40.39	12.82	79.37	20.84	103.87	11.16	123.60	18.84
0.0254	12.96	10.23	30.95	16.80	41.01	12.19	83.84	14.35	105.59	11.73	139.63	6.33
0.1	12.95	12.83	32.48	15.46	40.39	12.53	87.14	13.61	106.86	12.46	144.48	6.33
0.254	12.49	15.24	32.27	11.90	40.11	12.04	87.36	12.87	106.48	12.52	145.23	6.42
1	11.28	7.70	29.46	10.76	38.42	13.98	83.30	12.67	102.51	9.53	141.10	7.34
2	11.03	8.88	28.24	12.25	38.75	17.42	81.46	13.87	101.19	6.15	137.79	7.94
2.54	10.95	10.01	27.93	12.96	38.89	19.13	81.36	14.64	101.11	4.91	136.58	8.27
3	10.75	11.40	27.78	14.07	38.85	20.39	81.57	15.59	101.28	4.31	135.68	8.62
4	10.59	11.20	27.54	15.72	39.00	22.23	81.76	17.09	102.09	3.67	134.53	9.22
5	10.28	13.78	27.21	16.50	39.08	22.98	82.10	18.06	102.90	3.66	134.19	9.79
6	9.77	14.66	26.91	16.72	38.81	23.96	82.22	19.00	103.38	3.94	133.58	9.97
7	9.22	14.38	26.39	16.90	38.77	23.94	82.43	19.70	103.79	4.04	133.47	10.05
8	8.60	18.95	25.88	17.08	38.57	24.42	82.51	20.01	104.14	4.48	133.18	10.13
9	8.34	22.58	25.47	16.92	38.38	24.41	82.52	19.67	104.31	4.85	132.82	9.85
10	8.22	25.56	25.07	16.55	37.92	25.18	82.75	19.43	104.71	5.17	132.50	9.53
*F_max,ave_*	13.48	9.12	33.43	13.49	42.51	15.71	92.72	10.20	115.15	3.91	145.46	6.37

**Table 6:** Average values of force pushing the LWAC specimen off the non-pretensioned steel strand (*f_cm_* = 55.3 MPa).

*s* mm	*l_emb_*, mm											
	40		80		120		240		330		460	
	*F_ave_*, kN	CoV %	*F_ave_*, kN	CoV %	*F_ave_*, kN	CoV %	*F_ave_*, kN	CoV %	*F_ave_*, kN	CoV %	*F_ave_*, kN	CoV %
adhesion	10.87	42.78	29.17	29.35	43.44	34.02	85.70	9.33	109.83	10.00	151.89	11.38
0.01	12.94	37.56	33.68	22.27	48.17	25.68	94.07	8.29	112.21	9.87	155.11	8.64
0.0254	15.84	32.13	35.14	22.40	51.12	17.84	97.83	5.62	116.07	8.38	158.20	6.80
0.1	16.30	27.42	36.38	17.81	49.91	17.37	99.82	4.56	119.64	8.07	159.56	6.07
0.254	15.34	30.18	35.66	18.93	47.39	18.91	99.37	4.25	118.94	7.95	160.13	5.93
1	13.88	28.60	32.73	22.49	44.21	21.04	94.53	6.24	114.98	5.73	156.27	4.31
2	13.19	30.86	32.23	24.33	43.39	23.90	92.41	9.02	113.24	4.97	155.24	3.39
2.54	12.97	32.85	32.00	25.41	43.12	25.90	92.27	10.37	113.20	4.87	154.92	3.01
3	12.64	35.44	32.02	25.64	43.06	26.71	92.53	10.90	113.22	5.28	155.16	2.84
4	12.54	33.65	32.35	25.66	43.05	28.27	92.70	12.05	113.48	6.06	155.76	2.83
5	12.45	31.97	32.68	25.37	42.95	29.41	92.85	12.54	113.70	6.80	156.56	2.78
6	12.11	31.96	32.92	24.91	42.83	29.98	93.60	13.11	114.03	6.84	157.43	2.91
7	11.91	30.48	33.03	25.37	42.30	31.84	94.56	13.52	113.57	6.92	157.91	3.54
8	11.38	31.37	32.75	26.44	41.35	33.04	95.06	13.58	113.39	6.49	158.44	3.92
9	11.06	31.74	32.71	26.75	40.79	34.10	95.34	13.69	113.84	6.07	159.20	4.28
10	10.71	31.28	32.81	26.88	40.12	35.72	95.33	14.32	113.95	5.72	160.15	4.44
*F_max,ave_*	16.94	28.81	38.54	17.54	52.03	18.10	102.58	7.38	121.69	6.25	165.67	5.31

**Table 7:** Bond stress average values of LWAC specimen to non-pretensioned steel strand (*f_cm_* = 35.6 MPa).

*s* mm	*l_emb_*, mm											
	40		80		120		240		330		460	
	*f_b,ave_* MPa	CoV %	*f_b,ave_* MPa	CoV %	*f_b,ave_* MPa	CoV %	*f_b,ave_* MPa	CoV %	*f_b,ave_* MPa	CoV %	*f_b,ave_* MPa	CoV %
adhesion	3.16	20.57	4.00	20.52	4.09	13.86	4.43	22.84	3.96	19.08	3.75	21.61
0.01	3.51	14.82	4.77	21.80	4.47	12.08	4.72	19.06	4.01	18.20	3.80	19.31
0.0254	3.79	10.30	5.33	24.81	4.71	14.45	4.88	16.54	4.05	17.17	3.89	14.82
0.1	3.91	10.49	6.01	22.98	5.00	17.93	5.06	13.49	4.19	13.52	4.18	2.30
0.254	3.94	10.17	6.23	23.05	5.20	19.87	5.17	11.07	4.22	12.96	4.21	2.30
1	3.47	7.77	5.75	24.90	4.92	17.69	5.05	10.32	4.10	10.52	4.11	3.19
2	3.17	14.49	5.45	25.41	4.74	18.09	5.05	10.84	4.03	9.58	4.01	4.56
2.54	3.10	16.45	5.42	24.98	4.69	18.92	5.07	11.09	4.02	9.10	3.99	5.29
3	3.07	18.58	5.39	25.41	4.64	20.33	5.08	11.40	4.01	8.47	3.97	5.64
4	3.04	23.03	5.28	27.13	4.61	21.36	5.09	12.25	4.03	7.72	3.97	6.33
5	2.96	27.02	5.14	28.76	4.62	23.09	5.14	13.43	4.03	7.57	3.99	7.04
6	2.90	30.30	5.06	30.04	4.57	23.13	5.16	14.10	4.02	8.01	4.00	7.72
7	2.88	33.39	5.01	30.77	4.56	23.91	5.16	14.66	4.01	7.68	4.03	8.05
8	2.83	34.65	4.96	31.47	4.55	25.63	5.15	14.56	4.02	7.47	4.04	7.76
9	2.78	37.05	4.91	32.27	4.50	27.09	5.15	14.57	4.01	7.41	4.06	7.63
10	2.74	39.07	4.86	33.48	4.44	30.10	5.16	15.24	4.00	7.77	4.05	7.60
*f_b max,ave_*	4.05	11.85	6.24	23.08	5.23	19.70	5.34	8.87	4.42	9.96	4.26	3.29

**Table 8:** Bond stress average values of LWAC specimen to non-pretensioned steel strand (*f_cm_* = 45.2 MPa).

*s* mm	*l_emb_*, mm											
	40		80		120		240		330		460	
	*f_b,ave_* MPa	CoV %	*f_b,ave_* MPa	CoV %	*f_b,ave_* MPa	CoV %	*f_b,ave_* MPa	CoV %	*f_b,ave_* MPa	CoV %	*f_b,ave_* MPa	CoV %
adhesion	4.41	10.38	5.08	25.21	4.89	14.80	4.85	21.64	4.54	14.30	3.91	21.49
0.01	4.72	11.12	5.57	20.46	5.12	12.83	5.03	20.87	4.79	11.07	4.09	18.84
0.0254	4.93	10.23	5.89	16.82	5.20	12.19	5.31	14.30	4.87	11.71	4.62	6.28
0.1	4.92	12.84	6.18	15.54	5.12	12.52	5.52	13.58	4.93	12.38	4.78	6.28
0.254	4.75	15.24	6.14	11.90	5.09	12.04	5.54	12.82	4.91	12.43	4.80	6.45
1	4.29	7.69	5.60	10.71	4.87	13.98	5.28	12.69	4.73	9.52	4.67	7.29
2	4.20	8.87	5.37	12.29	4.91	17.42	5.16	13.94	4.67	6.22	4.56	7.90
2.54	4.16	10.02	5.31	12.99	4.93	19.13	5.16	14.73	4.66	4.93	4.52	8.19
3	4.09	11.40	5.28	14.01	4.92	20.39	5.17	15.66	4.67	4.28	4.49	8.69
4	4.03	11.19	5.24	15.66	4.94	22.24	5.18	17.17	4.71	3.61	4.45	9.22
5	3.91	13.78	5.17	16.43	4.95	22.99	5.20	18.06	4.74	3.58	4.44	9.69
6	3.72	14.63	5.12	16.80	4.92	23.96	5.21	18.99	4.77	3.99	4.42	9.96
7	3.51	14.37	5.02	16.94	4.92	23.95	5.23	19.71	4.78	3.97	4.41	9.97
8	3.27	18.94	4.92	17.07	4.89	24.42	5.23	20.08	4.80	4.58	4.40	10.22
9	3.17	22.56	4.84	16.93	4.87	24.41	5.23	19.69	4.81	4.78	4.39	9.79
10	3.13	25.57	4.77	16.57	4.81	25.19	5.25	19.44	4.83	5.18	4.38	9.58
*f_b max,ave_*	5.13	9.17	6.36	13.53	5.39	15.77	5.88	10.21	5.31	3.96	4.81	6.44

**Table 9:** Bond stress average values of LWAC specimen to non-pretensioned steel strand (fcm = 55.3 MPa).

*s* mm	*l_emb_*, mm											
	40		80		120		240		330		460	
	*f_b,ave_* MPa	CoV %	*f_b,ave_* MPa	CoV %	*f_b,ave_* MPa	CoV %	*f_b,ave_* MPa	CoV %	*f_b,ave_* MPa	CoV %	*f_b,ave_* MPa	CoV %
adhesion	4.13	42.86	5.55	29.37	5.51	34.12	5.43	9.21	5.06	10.08	5.02	11.35
0.01	4.92	37.60	6.40	22.19	6.11	25.70	5.96	8.39	5.17	9.86	5.13	8.58
0.0254	6.02	32.23	6.68	22.31	6.48	17.90	6.20	5.65	5.35	8.41	5.23	6.69
0.1	6.20	27.42	6.92	17.77	6.33	17.38	6.33	4.42	5.52	7.97	5.28	6.06
0.254	5.84	30.14	6.78	18.88	6.01	18.80	6.30	4.29	5.48	7.85	5.30	5.85
1	5.28	28.60	6.23	22.47	5.61	21.03	5.99	6.18	5.30	5.66	5.17	4.26
2	5.02	30.88	6.13	24.31	5.50	23.82	5.86	9.04	5.22	4.98	5.13	3.31
2.54	4.93	32.86	6.08	25.33	5.47	25.96	5.85	10.43	5.22	4.79	5.12	3.12
3	4.81	35.34	6.09	25.62	5.46	26.74	5.87	10.90	5.22	5.36	5.13	2.73
4	4.77	33.54	6.15	25.69	5.46	28.21	5.88	12.07	5.23	6.12	5.15	2.91
5	4.74	31.86	6.22	25.40	5.45	29.36	5.88	12.59	5.24	6.87	5.18	2.70
6	4.60	31.96	6.26	24.92	5.43	29.83	5.93	13.15	5.26	6.84	5.20	2.88
7	4.53	30.46	6.28	25.48	5.37	31.84	6.00	13.50	5.24	6.87	5.22	3.45
8	4.33	31.41	6.23	26.48	5.24	33.02	6.03	13.60	5.23	6.50	5.24	4.01
9	4.21	31.83	6.22	26.69	5.17	34.04	6.04	13.58	5.25	6.10	5.27	4.17
10	4.07	31.45	6.24	26.92	5.09	35.76	6.04	14.40	5.25	5.71	5.30	4.34
*f_b max,ave_*	6.44	28.88	7.33	17.60	6.60	18.03	6.50	7.38	5.61	6.24	5.48	5.29

**Table 10:** Average values of average bond stress for LWAC to plain seven-wire steel strand for specimens with *l_emb_* equal to 240, 330 and 460 mm.

*s* mm	*f_cm_* = 35.6 MPa		*f_cm_* = 45.2 MPa		*f_cm_* = 55.3 MPa	
	*f_b,ave_*, MPa	CoV %	*f_b,ave_*, MPa	CoV %	*f_b__,ave_*_,_ MPa	CoV %
adhesion	4.05	20.99	4.43	20.54	5.17	10.25
0.01	4.18	20.10	4.64	18.75	5.42	11.07
0.0254	4.27	18.50	4.93	12.58	5.59	10.20
0.1	4.48	14.29	5.08	12.60	5.71	9.98
0.254	4.53	13.91	5.08	12.40	5.69	9.67
1	4.42	13.35	4.89	11.25	5.49	8.56
2	4.36	14.22	4.80	11.25	5.40	8.70
2.54	4.36	14.68	4.78	11.51	5.40	9.07
3	4.35	14.94	4.78	12.13	5.41	9.43
4	4.36	15.37	4.78	12.97	5.42	9.96
5	4.39	15.95	4.80	13.54	5.43	10.13
6	4.40	16.36	4.80	14.17	5.46	10.62
7	4.40	16.59	4.81	14.55	5.49	11.11
8	4.40	16.14	4.81	14.97	5.50	11.27
9	4.41	16.33	4.81	14.76	5.52	11.23
10	4.40	16.59	4.82	14.73	5.53	11.39
*f_b,max,ave_* (MPa)	4.70	13.62	5.33	11.07	5.86	10.07
*f_b,ave_* (*s* = 1÷10) MPa	4.39		4.81		5.46	
*f_b,ave_* (*s* = 2÷10) MPa	4.38		4.80		5.46	

**Table 11:** Average values of the maximum force pushing the LWAC specimen off the non-pretensioned steel strand *F_max,ave_* (in kN).

*l_emb_*, mm	*f_cm_*, MPa		
	35.6	45.2	55.3
40	10.65	13.48	16.94
80	32.84	33.43	38.54
120	41.24	42.51	52.03
240	84.32	92.72	102.58
330	95.86	115.15	121.69
460	128.90	145.46	165.67

**Table 12:** Average values of the maximum bond stress of the LWAC specimen to non-pretensioned steel strand *f_bmax,ave_* (in MPa).

*l_emb_*, mm	*f_cm_*, MPa		
	35.6	42.5	55.3
40	4.05	5.13	6.44
80	6.24	6.36	7.33
120	5.23	5.39	6.60
240	5.34	5.88	6.50
330	4.42	5.31	5.61
460	4.26	4.81	5.48

**Table 13:** Average values of the maximum stress in the non-pretensioned steel strand *f_pmax,ave_* (in MPa).

*l_emb_*, mm	*f_cm_*, MPa		
	35.6	45.2	55.3
40	71.0	89.9	112.9
80	218.9	222.9	256.9
120	274.9	283.4	346.9
240	562.1	618.1	683.9
330	639.1	767.7	811.3
460	859.3	969.7	1104.5

**Table 14:** Ratio of real strand stress level to ultimate strength (*f_pmax,ave_*/*f_pk_*) for prestressing steel strand.

*l_emb_*_,_ mm	*f_cm_*, MPa		
	35.6	45.2	55.3
40	0.037	0.047	0.059
80	0.115	0.117	0.135
120	0.144	0.149	0.182
240	0.295	0.324	0.359
330	0.335	0.403	0.425
460	0.451	0.509	0.579

**Table 15:** Influence of the LWAC compressive strength and the square root of the LWAC compressive strength on the relative bond stress–slip relationship for the 15.7 mm steel strand, *l_emb_* = 240 mm.

*s*, mm	*f_b,ave_*/*f_cm_*					*f_b,ave_*/*√f_cm_*				
	*f_cm_*, MPa			Average	CoV %	*f_cm_*, MPa			Average	CoV %
	35	45	55			35	45	55		
adhesion	0.127	0.108	0.099	0.111	12.79	0.749	0.723	0.732	0.735	1.78
0.01	0.135	0.112	0.108	0.118	12.18	0.798	0.750	0.804	0.784	3.77
0.0254	0.139	0.118	0.113	0.123	11.46	0.825	0.792	0.836	0.818	2.83
0.1	0.145	0.123	0.115	0.127	12.01	0.855	0.823	0.854	0.844	2.16
0.254	0.148	0.123	0.115	0.129	13.40	0.874	0.826	0.850	0.850	2.83
1	0.144	0.117	0.109	0.124	14.96	0.854	0.787	0.808	0.816	4.17
2	0.144	0.115	0.107	0.122	16.30	0.854	0.769	0.790	0.804	5.46
2.54	0.145	0.115	0.106	0.122	16.61	0.857	0.769	0.789	0.805	5.72
3	0.145	0.115	0.107	0.122	16.55	0.859	0.771	0.792	0.807	5.70
4	0.145	0.115	0.107	0.123	16.57	0.860	0.772	0.793	0.809	5.70
5	0.147	0.116	0.107	0.123	17.07	0.869	0.775	0.793	0.812	6.13
6	0.147	0.116	0.108	0.124	16.94	0.872	0.777	0.800	0.816	6.11
7	0.147	0.116	0.109	0.124	16.41	0.872	0.780	0.809	0.820	5.77
8	0.147	0.116	0.110	0.124	16.11	0.871	0.780	0.813	0.821	5.60
9	0.147	0.116	0.110	0.124	16.05	0.871	0.780	0.814	0.822	5.58
10	0.147	0.117	0.110	0.125	16.07	0.872	0.783	0.814	0.823	5.52

**Table 16:** Influence of the LWAC compressive strength and the square root of the LWAC compressive strength on the relative bond stress–slip relationship for the 15.7 mm steel strand, *l_emb_* = 330 mm.

*s* mm	*f_b,ave_*/*f_cm_*					*f_b,ave_*/*√f_cm_*				
	*f_cm_*, MPa			Average	CoV %	*f_cm_*, MPa			Average	CoV %
	35	45	55			35	45	55		
adhesion	0.113	0.101	0.092	0.102	10.41	0.669	0.677	0.682	0.676	0.96
0.01	0.115	0.106	0.094	0.105	9.87	0.678	0.714	0.697	0.696	2.60
0.0254	0.116	0.108	0.097	0.107	8.66	0.685	0.726	0.721	0.711	3.19
0.1	0.120	0.110	0.100	0.110	8.81	0.708	0.735	0.744	0.729	2.57
0.254	0.121	0.109	0.100	0.110	9.55	0.713	0.732	0.739	0.728	1.82
1	0.117	0.105	0.096	0.106	9.82	0.693	0.705	0.715	0.704	1.54
2	0.115	0.104	0.095	0.105	9.70	0.681	0.696	0.704	0.694	1.66
2.54	0.115	0.104	0.095	0.104	9.58	0.680	0.695	0.704	0.693	1.78
3	0.115	0.104	0.095	0.104	9.43	0.678	0.696	0.704	0.693	1.93
4	0.115	0.105	0.095	0.105	9.55	0.681	0.702	0.705	0.696	1.88
5	0.115	0.105	0.095	0.105	9.44	0.681	0.707	0.707	0.698	2.10
6	0.115	0.106	0.096	0.106	9.12	0.680	0.711	0.709	0.700	2.53
7	0.115	0.106	0.095	0.105	9.19	0.678	0.713	0.707	0.699	2.66
8	0.115	0.107	0.095	0.106	9.41	0.680	0.716	0.705	0.700	2.65
9	0.115	0.107	0.096	0.106	9.11	0.678	0.717	0.708	0.701	2.93
10	0.114	0.107	0.096	0.106	9.01	0.676	0.720	0.708	0.701	3.23

**Table 17:** Influence of the LWAC compressive strength and the square root of the LWAC compressive strength on the relative bond stress–slip relationship for the 15.7 mm steel strand, *l_emb_* = 460 mm.

*s* mm	*f_b,ave_*/*f_cm_*					*f_b,ave_*/√*f_cm_*				
	*f_cm_*, MPa			Average	CoV %	*f_cm_*, MPa			Average	CoV %
	35	45	55			35	45	55		
adhesion	0.107	0.087	0.091	0.095	11.20	0.634	0.583	0.677	0.631	7.46
0.01	0.109	0.091	0.093	0.098	9.83	0.642	0.610	0.692	0.648	6.37
0.0254	0.111	0.103	0.095	0.103	7.80	0.658	0.689	0.705	0.684	3.54
0.1	0.119	0.106	0.096	0.107	10.96	0.707	0.713	0.712	0.710	0.47
0.254	0.120	0.107	0.096	0.108	11.13	0.712	0.716	0.715	0.714	0.29
1	0.117	0.104	0.094	0.105	11.20	0.695	0.696	0.697	0.696	0.17
2	0.115	0.101	0.093	0.103	10.43	0.678	0.680	0.692	0.683	1.10
2.54	0.114	0.100	0.093	0.103	10.26	0.674	0.674	0.692	0.680	1.50
3	0.113	0.100	0.093	0.102	10.07	0.671	0.669	0.692	0.677	1.84
4	0.113	0.099	0.094	0.102	10.05	0.671	0.663	0.694	0.676	2.39
5	0.114	0.099	0.094	0.102	10.16	0.674	0.662	0.699	0.678	2.74
6	0.114	0.098	0.095	0.102	10.26	0.676	0.659	0.701	0.679	3.13
7	0.115	0.098	0.095	0.103	10.61	0.681	0.657	0.704	0.681	3.41
8	0.115	0.098	0.095	0.103	10.68	0.683	0.656	0.707	0.682	3.72
9	0.116	0.098	0.096	0.103	10.85	0.686	0.654	0.711	0.684	4.12
10	0.116	0.097	0.096	0.103	10.57	0.685	0.653	0.715	0.684	4.51

## Data Availability

The original contributions presented in the study are included in the article, further inquiries can be directed to the corresponding author.
